# IPF-LASSO: Integrative *L*_1_-Penalized Regression with Penalty Factors for Prediction Based on Multi-Omics Data

**DOI:** 10.1155/2017/7691937

**Published:** 2017-05-04

**Authors:** Anne-Laure Boulesteix, Riccardo De Bin, Xiaoyu Jiang, Mathias Fuchs

**Affiliations:** ^1^Department of Medical Informatics, Biometry and Epidemiology, University of Munich (LMU), Marchioninistr. 15, 81377 Munich, Germany; ^2^Department of Mathematics, University of Oslo, Moltke Moes Vei 3, 0851 Oslo, Norway; ^3^Novartis Institutes for BioMedical Research, 250 Massachusetts Avenue, Cambridge, MA 02139, USA; ^4^Biogen, 225 Binney Street, Cambridge, MA 02142, USA

## Abstract

As modern biotechnologies advance, it has become increasingly frequent that different modalities of high-dimensional molecular data (termed “omics” data in this paper), such as gene expression, methylation, and copy number, are collected from the same patient cohort to predict the clinical outcome. While prediction based on omics data has been widely studied in the last fifteen years, little has been done in the statistical literature on the integration of multiple omics modalities to select a subset of variables for prediction, which is a critical task in personalized medicine. In this paper, we propose a simple penalized regression method to address this problem by assigning different penalty factors to different data modalities for feature selection and prediction. The penalty factors can be chosen in a fully data-driven fashion by cross-validation or by taking practical considerations into account. In simulation studies, we compare the prediction performance of our approach, called IPF-LASSO (Integrative LASSO with Penalty Factors) and implemented in the R package ipflasso, with the standard LASSO and sparse group LASSO. The use of IPF-LASSO is also illustrated through applications to two real-life cancer datasets. All data and codes are available on the companion website to ensure reproducibility.

## 1. Introduction 

Most drugs cannot treat all patients with a given disease. It is thus crucial to identify biomarkers (genetic, genomic, proteomic, or any measurable biological entities) that can predict the patient's response to a given therapy. Ultimately, the biomarkers are to be built into companion diagnostic kits. Ideally, the number of biomarkers should be small to reduce the labor and cost.

High-throughput molecular data, termed “omics data” in this paper, have been used for developing prediction models for more than fifteen years. As a well-known example, gene expression data have often been found to be useful for predicting survival response to therapy of cancer patients; the overwhelming enthusiasm in the initial years has meanwhile been tempered by more critical studies [1]. In the last few years, bioassay technology improvement and cost reduction have made collecting several types of high-dimensional data in the same study feasible.

For example, methylation data, copy number data, and mRNA expression may be available for the same patient cohort. Other data types include microRNA expression, proteomic data, metabolomic data, and single nucleotide polymorphisms (SNPs). In this paper, we denote each group of variables of the same type as a “modality” and the whole dataset as a “multi-omics” dataset. For example, in this paper, we consider as illustration a breast cancer dataset with a clinical modality and a gene expression modality [2] and a leukemia dataset from The Cancer Genome Atlas [3] with a clinical modality, a gene expression modality, and a copy number variation modality.

As multiple modalities of biomarker measurements become available for the same patients, the research interest starts to focus on the integration of data modalities to identify biomarkers and build prediction models with good accuracy [4, 5]. Although using omic markers for prediction has been a well-studied topic, it is not clear how the different modalities should be handled. The most straightforward yet naive approach is to merge all datasets and ignore the source of the variables. In contrast, other authors suggest analyzing each modality on its own and then merging the results [6], whereby merging can be performed at different stages of the analysis [7]. However, the literature is often vague on when to use different strategies.

The case of variables from one low-dimensional modality (typically, a few clinical variables relevant to the outcome to be predicted) and one high-dimensional modality (e.g., a microarray gene expression dataset) has been extensively investigated by De Bin et al. [8], where they assess the “residual” two-step approach and the “favoring” approach (see Section 2.2 for more details).

There has been a large amount of statistical and bioinformatic literature on the integration of multiple omics datasets investigating their correlation structure [9]. However, the focus of these works is not prediction. Our motivation here is to suggest a simple method based on a well-investigated framework, which takes into account the data modalities while integrating them into a sparse prediction model. Our method is based on *L*_1_-penalized regression (LASSO) [10] and takes the data structure into account by assigning different penalty factors to the modalities. The penalty factors are either determined by cross-validation or prespecified by the user. We name this method IPF-LASSO (Integrative LASSO with Penalty Factors).

In simulation studies, we show that IPF-LASSO performs better than the standard LASSO when the proportions of relevant variables are different in different modalities and generates parsimonious prediction rules compared with sparse group LASSO. An R package called ipflasso implementing this method is made publicly available on R/CRAN website. Being directly based on LASSO, our approach has two major advantages: its conceptual simplicity within a well-established framework and its* transportability* [11] (e.g., in the case of binary outcomes, users only need to know the fitted regression coefficients to apply the prediction rule).

This paper is structured as follows. After a short introduction into *L*_1_-penalized regression, the newly proposed method is described in detail in Section 2. The results from simulation studies and two real-life applications are presented in Sections 3 and 4, respectively. All data and codes are available on http://www.ibe.med.uni-muenchen.de/organisation/mitarbeiter/020_professuren/boulesteix/ipflasso/ to ensure reproducibility.

## 2. Methods

### 2.1. IPF-LASSO

#### 2.1.1. Principle

We denote the standardized predictor variable *j* measured from subject *i* as *x*_*ij*_ and the centered (continuous) response values as *y*_*i*_, where *i* = 1,…, *n* and *j* = 1,…, *p*. The standard LASSO method [10] solves the *L*_1_-penalized regression problem by finding **β** = {*β*_*j*_} which minimizes ∑_*i*=1_^*n*^(*y*_*i*_ − ∑_*j*=1_^*p*^ *x*_*ij*_ *β*_*j*_)^2^ + *λ*∑_*j*=1_^*p*^|*β*_*j*_| = ∑_*i*=1_^*n*^(*y*_*i*_ − ∑_*j*=1_^*p*^ *x*_*ij*_ *β*_*j*_)^2^ + *λ*‖**β**‖_1_, where ‖·‖_1_ denotes the *L*_1_-norm. The *L*_1_-penalty shrinks some of the coefficients to 0, thus leading to an intrinsic variable selection. For a historical overview of the development of LASSO regression and some variations, readers can refer to Tibshirani [12].

This framework can be generalized to logistic regression (in the case of a binary outcome) and to Cox proportional hazards regression (in the case of a censored time to event). The term ∑_*i*=1_^*n*^(*y*_*i*_ − ∑_*j*_ *x*_*ij*_ *β*_*j*_)^2^ is replaced by −*ℓ*(**β**, *γ*) (where *ℓ*(·, ·) stands for the log-likelihood function and *γ* for the intercept) in the logistic LASSO and is replaced by −*pℓ*(**β**) (where *pℓ*(·) stands for the partial log-likelihood) in the Cox LASSO. Our new method is a modification of LASSO dedicated to the case where multiple data modalities (data types) from the same subjects are to be used. Let us denote the variables from modality *m* (for *m* = 1,…, *M*) as *X*_1_^(*m*)^,…, *X*_*p*_*m*__^(*m*)^ and their values for subject *i* (for *i* = 1,…, *n*) as *x*_*i*1_^(*m*)^,…, *x*_*ip*_*m*__^(*m*)^, where *p*_*m*_ is the number of variables from modality *m*. Similarly, *β*_*j*_^(*m*)^ denotes the coefficient of variable *X*_*j*_^(*m*)^.

We propose the use of a weighted sum of the *L*_1_ norms of the coefficient vectors of each modality **β** ^(*m*)^ = (*β*_1_^(*m*)^,…,*β*_*p*_*m*__^(*m*)^)^*⊤*^ (*m* = 1,…, *M*) as the penalty term, aiming to account for their different relevancies. In our method, the estimated coefficients are those that minimize

(1) $$\begin{array}{c} \overset{n}{\underset{i=1}{\sum }}{\left(\;y_{i}-\sum_{m=1\;}^{M}\sum_{j=1}^{p_{m}}x_{ij}^{\left(m\right)}\beta_{j}^{\left(m\right)}\;\right)}^{2}+\overset{M}{\underset{m=1}{\sum }}\lambda_{m}{\left\|\;\beta^{\left(m\right)}\;\right\|}_{1}, \end{array}$$

where *λ*_*m*_ > 0 is the penalty applied to the variables from modality *m*. We call this method “IPF-LASSO,” standing for Integrative LASSO with Penalty Factors. The term “penalty factors” refers to the multiplicative factors applied to the penalty term. Without restriction of generality, we consider the first modality as reference modality—with penalty *λ*_1_ and penalty factor 1—and define the penalty factor of modality *m* as *λ*_*m*_/*λ*_1_.

Similar to the standard LASSO, our proposed framework can be applied to *L*_1_-penalized regression with linear, binary, or time-to-event outcomes. The rationale of the penalty term given in (1) is that in reality the proportion of relevant variables is often highly different from one modality to another; hence, it makes sense to penalize the modalities differently.

The Bayesian interpretation of the LASSO is useful to outline the motivation of the different penalty parameters. Park and Casella [13] show that the LASSO estimate for linear regression parameters can be interpreted as a Bayesian posterior mode estimate when the regression parameters have independent Laplace (i.e., double-exponential) priors. In this perspective, using different penalties for different modalities amounts to setting different parameters for the Laplace priors. It can be seen as a way of using the available prior information to improve the estimation of coefficients and, ultimately, prediction accuracy.

Note that our approach may also be seen as connected with the adaptive LASSO [14], in the sense that the coefficients of variables that are identified as informative are less penalized than the coefficients of noninformative variables. However, in contrast to adaptive LASSO [14], this modification of the penalty strength does not happen through a first LASSO step for each variable individually but at the level of the whole modality.

#### 2.1.2. Estimation

From a computational point of view, IPF-LASSO with fixed penalty factors is not more complex than the respective form of LASSO (linear, logistic, or Cox) with the same penalty for all variables, in that estimates can be simply obtained with any standard LASSO algorithm by preliminarily scaling the variables using their respective penalty. More precisely, the standard estimation algorithm is run with the same penalty parameter *λ*_1_ for all variables on the transformed data *x*_*ij*_^(*m*)*∗*^ = *x*_*ij*_^(*m*)^/(*λ*_*m*_/*λ*_1_)  (*i* = 1,…, *n*, *j* = 1,…, *p*). Estimates ${\hat{\beta }}_{j}^{(m)*}$ are obtained and rescaled as ${\hat{\beta }}_{j}^{\left(m\right)}={\hat{\beta }}_{j}^{(m)*}/(\lambda_{m}/\lambda_{1})$ to obtain the IPF-LASSO estimates.

### 2.2. Connections between IPF-LASSO and Other LASSO Variations for Omics Data

There have been LASSO variations for single and multiple data modalities proposed by several groups. In this section, we discuss the connections of IPF-LASSO to these methods. In the scenario investigated by De Bin et al. [8], we have two modalities (*M* = 2). The first modality includes only a small number *p*_1_ of clinical variables, such that a classical regression approach can be applied to this modality (the rule of thumb that the number of variables times 5 or 10 should not exceed the number of observations is typically satisfied). The second modality is high-dimensional with *p*_2_ ≫ *n*. In this case, it is sensible to penalize only the second modality, that is, to consider the penalty term *λ*‖**β** ^(2)^‖_1_. In the terminology of De Bin et al. [8], the above is denoted as a “favoring” method, because the smaller clinical modality is not penalized; in other words, it is “favored.” Another method, namely, the “residual” method as proposed in De Bin et al. [8], takes two steps fitting the data. First a classical (linear, logistic, or Cox) regression is fit to the first modality to estimate *β*_1_^(1)^,…, *β*_*p*_1__^(1)^; the resulting linear predictor ${\hat{\beta }}_{1}^{\left(1\right)}x_{i1}^{\left(1\right)}+\cdots +{\hat{\beta }}_{p_{1}}^{\left(1\right)}x_{ip_{1}}^{\left(1\right)}$ is then considered as an offset during the estimation of *β*_1_^(2)^,…, *β*_*p*_2__^(2)^ through LASSO regression. These two methods, however, cannot be applied when there are multiple high-dimensional modalities because it would not be feasible to estimate the coefficients. Moreover, they may lead to a decrease of accuracy if the favored modality is in reality not the most relevant for prediction.

Another two-step approach for prediction is proposed by Zhao et al. [6]: they first apply LASSO regression to multi-omics data to select a small number (10 in their application) of variables from each modality and then use the selected variables in a *L*_2_-penalized Cox regression model. This approach does not take correlations between variables from different modalities into account.

Group LASSO [15, 16] and sparse group LASSO [17] represent another category of LASSO extensions for data with a group structure. In the case of multiple modalities, the term “group” is essentially “modality.” The principle of group LASSO is that variables from the same group should be either all selected or all discarded. It makes sense, for example, when each group consists of the dummy variables coding the same multicategorical variable. The penalty considered in the group LASSO method has the form $\lambda \sum_{m=1}^{M}\sqrt{p_{m}}{\left\|\beta^{\left(m\right)}\right\|}_{2}.$ With multiple large omics modalities considered in our paper, it is most likely that at most a few variables from each modality are truly relevant for prediction; hence, this “none-versus-all” assumption is not reasonable in this case.

Sparse group LASSO [17] relaxes the “none-versus-all” assumption by introducing some sparsity within groups. This is achieved by combining the penalty of group LASSO with an *L*_1_ penalty, yielding the penalty term $(1-\alpha )\lambda \sum_{m=1}^{M}\sqrt{p_{m}}{\left\|\beta^{\left(m\right)}\right\|}_{2}+\alpha \lambda {\left\|\beta \right\|}_{1}$, where *α* is a so-called “mixing parameter” comprised between 0 and 1. Sparse group LASSO can be used in cases where IPF-LASSO is aimed at addressing where a modality is treated as a group. However, these two methods are fundamentally different. In sparse group LASSO, a single mixing parameter *α* balances the impact of group structure and overall sparsity; thus, a model that strongly reflects the group structure is obtained at the price of reduced sparsity; moreover, the degree of *L*_1_ shrinkage is the same for all groups (modalities) as controlled by *α*, which often does not reflect reality. IPF-LASSO, on the other hand, is more flexible in varying the *L*_1_ shrinkage parameters for different modalities—at the price of more tuning parameters (one for each modality) in the case of more than two modalities. In Section 3, we will compare the performances of IPF-LASSO and sparse group LASSO.

Another recently proposed approach handling two modalities in the framework of penalized regression is collaborative regression [18]. The idea is using a penalty not only based on the *L*_1_- or *L*_2_-norms of the coefficients but also penalizing the difference between the fitted linear predictors resulting from each of the two modalities. The two modalities “collaborate” in the sense that they are forced to yield similar contributions to prediction. In the mathematical terms and adapted to our notation, the penalty term considered in Gross and Tibshirani [18] is ‖**X** ^(1)^ **β** ^(1)^ − **X** ^(2)^ **β** ^(2)^‖^2^ + *P*_1_(**β** ^(1)^) + *P*_2_(**β** ^(2)^), where *P*(·) is the general notation for a penalty term that can, for example, be based on the *L*_1_- or *L*_2_-norm and **X** ^(*m*)^ denotes the *n* × *p*_*m*_ data matrix for modality *m* (with *m* = 1,2 here). Note that Gross and Tibshirani [18] state that this method is not well suited for prediction but rather finds common patterns shared by the two modalities by forcing the fitted linear predictors from each modality to be similar.

For the sake of exhaustiveness, let us also mention an applied paper on plant breeding [19] using the idea of applying different penalties to variables from two different modalities (genetic markers and metabolomic traits in their case), however, in the different context of ridge regression (i.e., *L*_2_-penalty as opposed to LASSO) for a continuous outcome. In their study, published in a genetics journal and focusing on the agricultural application, they apply this method to their dataset and do not investigate it from a methodological point of view. A similar approach based on *L*_2_-penalized logistic regression [20] formalizes and extends this idea with the purpose to better integrate external data such as annotation or external *p* values.

In summary, the IPF-LASSO proposed here is aimed at using multiple high-dimensional data modalities in a flexible way by weighing them differently in feature selection and prediction modelling, which is a critical yet unsolved problem in biomedical research.

### 2.3. Cross-Validation for the Choice of the Penalty Parameters

In this section, we discuss the choice of the parameters *λ*_1_,…, *λ*_*m*_. Similar to *λ* in the case of the standard LASSO, values for the penalty factors *λ*_1_, *λ*_2_/*λ*_1_,…, *λ*_*M*_/*λ*_1_ can be determined by cross-validation (CV) based on prediction performance. In our study, we use 5-fold CV with 10 repeats as a good compromise between performance and computation time [21]. Common metrics quantifying prediction performance include the mean squared error for continuous outcomes, the misclassification rate (or 1 − accuracy), the area under the ROC curve (AUC) for binary outcomes, or the partial likelihood for time-to-event outcomes. In practice, we implement the procedure as follows. We consider *C* different candidate vectors of penalty factors of the form **s** ^(*c*)^ = (1, *λ*_2_/*λ*_1_,…,*λ*_*M*_/*λ*_1_)^*⊤*^, with *c* = 1,…, *C*; for each candidate vector **s** ^(*c*)^ of penalty factors, we apply CV with the chosen performance metric to select the optimal *λ*_1_; the vector **s** ^(*c*_opt_)^ of penalty factors whose optimal *λ*_1_ yields the best fit according to the chosen performance metric is finally selected.

### 2.4. Software Implementation

IPF-LASSO is implemented in our new R package ipflasso, which is publicly available from the CRAN. It is based on the R package glmnet and includes the following features and improvements.

*Rescaling Procedure*. The rescaling procedure described in Section 2.1.2 is implemented in the R package glmnet [22] through the argument penalty.factor. This argument has the form penalty.factor=c(rep(1,p1),...,rep(pfM,pM)) in IPF-LASSO, where p1,…,pM are the sizes of the *M* modalities and pfM stands for *λ*_*M*_/*λ*_1_ (note that the result is invariant against multiplication of the vector of penalty factors by a scalar). The functions from our package ipflasso automatically generate the argument penalty.factor when given the indices of the variables from each modality and the values *λ*_*m*_/*λ*_1_ (*m* = 2,…, *M*).

*Cross-Validation for the Choice of λ*_1_. For a fixed set of penalty factors, *λ*_1_ can be selected using the function cv.glmnet from package glmnet [22]. However, cv.glmnet cannot perform repeated CV in its current version. An extended version of cv.glmnet allowing repetition of CV is implemented in the function cvr.glmnet from our package ipflasso. Repeated CV applied in combination with penalty factors for variables from different modalities is implemented in the function cvr.ipflasso.

*Cross-Validation for the Choice of Penalty Factors*. Finally, the R package ipflasso also includes a function, cvr2.ipflasso, to perform CV in the two dimensions of the grid: choice of *λ*_1_ for fixed penalty factors and choice of the penalty factors *λ*_*m*_/*λ*_1_ (*m* = 2,…, *M*). It takes the candidate sets of penalty factors **s** ^(1)^,…, **s** ^(*C*)^ as an argument. The function cvr2.ipflasso allows one to set a maximal number of variables to be included in the final model. The CV-based choice of the parameters is then performed only over values yielding models of this size or sparser.

As an example, the following simple code performs 5-fold cross-validation repeated 10 times to choose the best penalty factors out of **s** ^(1)^ = (1,1), **s** ^(12)^ = (1,2), **s** ^(3)^ = (1,4), **s** ^(4)^ = (1, 1/2), and **s** ^(5)^ = (1, 1/4), where the 200 predictor variables come from two modalities (one consisting of the 50 first variables and the other consisting of the 150 last variables). 

> X<-matrix (rnorm(50*∗*200),50,200)

> Y<-rbinom (50,1,0.5)

> cvr2.ipflasso(X=X,Y=Y,

family="binomial",type.measure="class",

standardize=TRUE, blocks=list (block1=1 : 50,block2=51 : 200),

pflist=list(c(1,1),c(1,2),c(2,1),c(1,4),c(4,1)),nfolds=5,ncv=10)

The criteria used for cross-validation currently implemented in ipflasso are the mean squared error for continuous outcomes, the misclassification rate or the area under curve (AUC) for binary outcomes, and the partial likelihood for time-to-event outcomes.

## 3. Simulations

### 3.1. Simulation Design

The goal of simulation studies is to investigate the performance of IPF-LASSO and compare it with other methods. We consider a binary dependent variable and two high-dimensional data modalities. The two modalities of variables vary in (i) their total numbers of variables *p*_1_ and *p*_2_, (ii) their numbers of truly relevant variables *p*_1_^*r*^ ≤ *p*_1_ and *p*_2_^*r*^ ≤ *p*_2_, and (iii) the effects *β*_1_ and *β*_2_ of the relevant variables. In all settings, *B* = 100 datasets of size *n* = 100 are successively randomly generated as follows. The binary class is drawn from the Bernoulli distribution with probability of success *τ* = 0.5. The variables are then drawn from the multivariate normal distributions:

(2) X1,…,Xp1+p2 ∣ Y=0~MN0p1+p2,Σ,X1,…,Xp1+p2 ∣ Y=1~MNμ,Σ,

where the (*p*_1_ + *p*_2_)×(*p*_1_ + *p*_2_) covariance matrix Σ is set to the identity matrix **I**_*p*_1_+*p*_2__ in the main design and the mean vector ***μ*** is given as

(3) $$\begin{array}{c} \mu^{⊤}=\left(\;\underset{p_{1}^{r}}{\underset{︸}{\beta_{1},\ldots ,\beta_{1}}},0,\ldots ,0,\underset{p_{2}^{r}}{\underset{︸}{\beta_{2},\ldots ,\beta_{2}}},0,\ldots ,0\;\right). \end{array}$$

In the main design, we consider the settings (i.e., combinations of *p*_1_, *p*_2_, *p*_1_^*r*^, *p*_2_^*r*^, *β*_1_, and *β*_2_) displayed in Table 1. Setting A reflects the unrealistic situation of two modalities that are perfectly identical in terms of size (*p*_1_ = *p*_2_ = 1000), number/proportion of relevant variables (*p*_1_^*r*^ = *p*_2_^*r*^ = 10), and effects (*β*_1_ = *β*_2_ = 0.5). In setting B, the proportions of truly relevant variables are the same in both modalities (*p*_1_^*r*^/*p*_1_ = *p*_2_^*r*^/*p*_2_ = 0.03) and their effects are also equal, but modality 1 is much smaller (*p*_1_ = 100) than modality 2 (*p*_2_ = 1000). In setting C, the sizes of the modalities are as in setting B and the effects are also equal, but the numbers of truly relevant variables (*p*_1_^*r*^ = *p*_2_^*r*^ = 10) are such that the* proportions* of truly relevant variables are different in the two modalities (*p*_1_^*r*^/*p*_1_ = 0.1 versus *p*_2_^*r*^/*p*_2_ = 0.01). This difference is more pronounced in setting D: the proportions are 0.20 for modality 1 and 0 for modality 2, a quite common situation in practice (“useless omics data”). Setting E also reflects a common situation: the small modality 1 (*p*_1_ = 20) contains *p*_1_^*r*^ = 3 strong predictors (*β*_1_ = 1), which is, for instance, often the case of clinical variables or a small hypothesis-driven biomarker panel. In contrast, the large modality 2 (*p*_2_ = 1000) contains *p*_1_^*r*^ = 10 weak predictor variables (*β*_2_ = 0.3). Finally, in setting F, the sizes of the modalities are the same as those in setting E but there are more truly relevant variables in modality 1 (*p*_1_^*r*^ = 15) and less ones in modality 2 (*p*_2_^*r*^ = 3), and their effects are equal (*β*_1_ = *β*_2_ = 0.5). This situation, which is intermediate between settings D and E, is also common in practice.

For all *B* = 100 datasets within each of the six settings (A–F), we derive prediction models using four different methods.

*IPF*. Our method IPF-LASSO is applied, with candidate penalty factors (1, 2^*k*^) for *k* = −3, −2, −1,0, 1,2, 3. Note that when *k* = 0, that is, when *λ*_1_ = *λ*_2_, the method is equivalent to the standard LASSO. A 5-fold CV with 10 repeats is used. The criterion used in CV for selecting *λ* is the misclassification rate. All the other parameters of the penalized regression algorithm are set to the default values of the package glmnet.

*Standard*. The standard LASSO, that is, the modality structure, is ignored. This is equivalent to IPF-LASSO with penalty factors (1,1) as unique candidate. The parameters are the same as for IPF.

*SGL*. The sparse group LASSO [17] is as implemented in the R package SGL [23]. A 5-fold CV with no repeat is used as the repeat option is not available in SGL. All parameters are set to the default values of the package SGL except for thres, which is set to 0.01 instead of 0.001 to keep the computational time comparable with the other methods (our tests suggested that the resulting loss of accuracy is minimal).

*S*. Separate models are fitted successively using the standard LASSO. A 5-fold CV with 10 repeats is used to determine the parameter *λ*. The two resulting linear predictors are then combined through a logistic regression model for prediction.

In each simulation setting, prediction performance of all fitted models is evaluated through an independently drawn test dataset of size *n*_test_ = 5000. The misclassification rate and the area under curve (AUC) are computed with this test set for comparison of the methods. Additionally, we also depict (i) which penalty factor was selected by the cross-validation procedure for IPF-LASSO and (ii) the number of selected variables for all methods: IPF-LASSO, standard LASSO, sparse group LASSO, and S.

Note that simulation results are strongly dependent on the parameters and many other parameter settings are conceivable. To gain a better idea of our method's behavior, we additionally consider a total of 33 other simulation scenarios, results from which are presented in a more compact form. These additional parameter settings are displayed in Supplementary Table  1 (in Supplementary Material available online at https://doi.org/10.1155/2017/7691937).

In real life, variables may be correlated both within and across modalities due to biological relationship. To investigate whether correlation structure affects the method's behavior, we additionally consider settings, denoted as A′ to F′, based on settings A to F where a nondiagonal covariance matrix Σ is used instead of **I**_*p*_1_+*p*_2__.

More specifically, we assume that each modality contains *b* = 10 groups of mutually correlated variables, corresponding to a block diagonal covariance matrix within each modality. Moreover, we assume correlation between the variables from the *j*th group in modality 1 and the variables from the *j*th group in modality 2. In our study, we consider correlations of *ρ* = 0.4 and use the (*p*_1_ + *p*_2_)×(*p*_1_ + *p*_2_) covariance matrix Σ given as
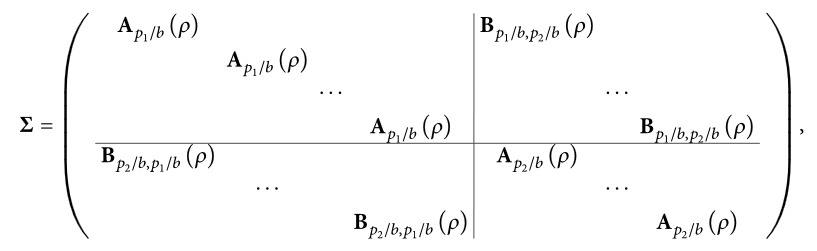
(4)where all empty entries are zero, **A**_*q*_(*ρ*) (with *q* = *p*_1_/*b* or *q* = *p*_2_/*b*) is the (*q* × *q*) matrix with ones on the diagonal and *ρ* outside of the diagonal, and **B**_*q*_1_,*q*_2__(*ρ*) (with *q*_1_ = *p*_1_/*b* and *q*_2_ = *p*_2_/*b* or vice versa) is the (*q*_1_ × *q*_2_) matrix with all entries equalling *ρ*. After generating the data from this multivariate normal distribution, we randomly permute the columns (i.e., the ordering of the variables), so that the informative variables (corresponding to the first—nonzero—entries of the vector ***μ***) are randomly distributed over the *b* blocks.

### 3.2. Simulation Results

#### 3.2.1. Main Simulation Results

**Figure 1:**
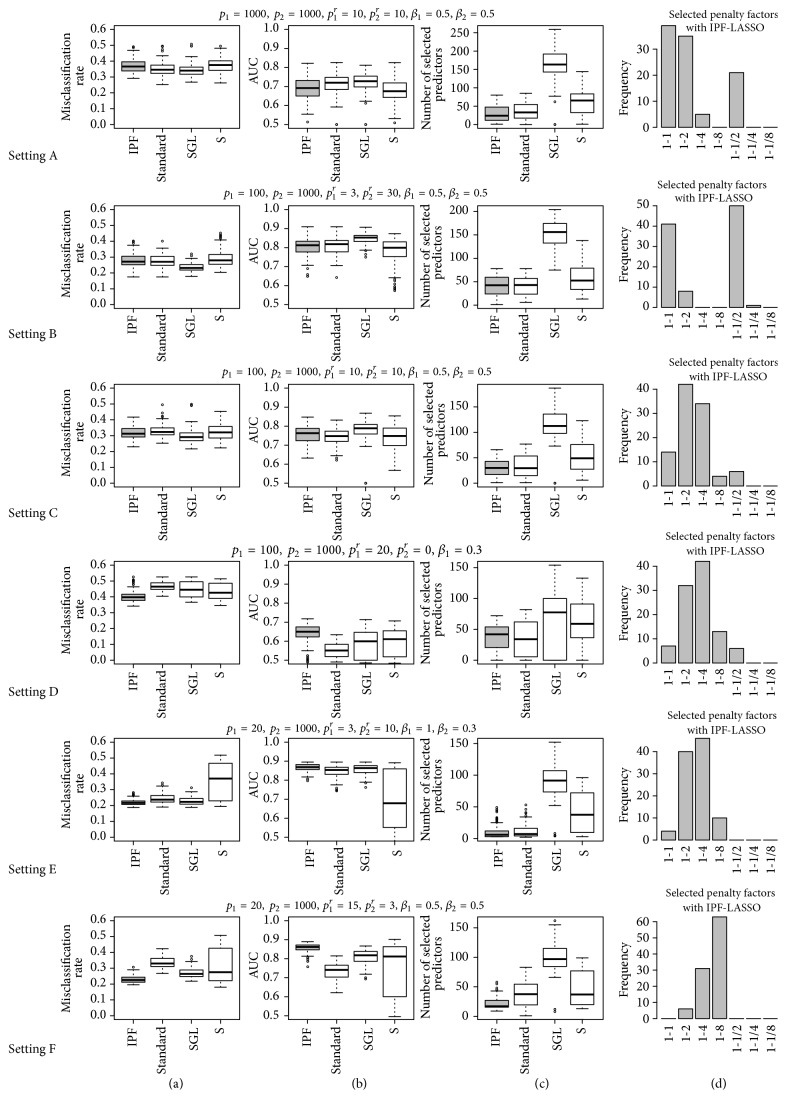
Results for settings A to F: misclassification rate on test set (a), AUC on test set (b), number of selected variables (c), and penalty factors selected by IPF (d).

Figure 1 displays the results for settings A to F. Figures 1(a) and 1(b) show misclassification rate and AUC (*y*-axis) for different methods (*x*-axis); Figure 1(c) shows the numbers of the selected variables and Figure 1(d) displays the penalty factors selected by cross-validation by IPF-LASSO.

Sparse group LASSO (SGL) performs better in terms of misclassification rate and AUC than IPF-LASSO in setting A where the two modalities are identical, in setting B where the proportions of truly relevant variables are the same, and in setting C where the number of truly relevant variables are the same. This observation indicates that when the two modalities are very similar, SGL tends to produce models with higher prediction performance.

Importantly, we notice that the improved prediction performance of SGL over IPF-LASSO in this case comes at a price of selecting substantially more variables into the final model, as shown in Figure 1(c). For example, in setting A, IPF-LASSO selects 24 variables (median over 100 simulation runs) whereas SGL selects more than 150 variables; in settings B and C, the numbers of the selected variables for SGL are above 100. This can be a major inconvenience in reality when both model size and prediction performance are relevant. For instance, when developing a companion diagnostic kit using biomarkers to predict patients' response to treatment, having a small set of around 10 markers is preferable to having over 100, from the point of view of cost- and labor-efficiency. Therefore, although the accuracy of IPF-LASSO is lower in some cases, it might still be more useful than SGL considering the overall practical utility. The tuning parameter *α* in SGL can be adjusted to change the sparsity; however, fine-tuning this parameter requires one more layer of cross-validation (and a large increase of computation time) and is out of the scope in this paper. Note that such a cross-validation is not implemented in the package SGL, suggesting that it is not particularly recommended by the authors.

In settings A, B, and C, the performance of the standard LASSO is slightly superior to IPF-LASSO. It makes sense in that when two data modalities are equally informative, giving them the same penalty is expected to yield better results than penalizing them differently. Due to the variability of cross-validation, however, IPF-LASSO does not always recognize that the best penalty factors are (1,1), leading to a slightly worse prediction performance.

In settings D, E, and F where two modalities are very different in the proportions of truly relevant variables, IPF-LASSO yields a better performance than the standard LASSO and SGL. When there is a belief that one modality is more relevant to the outcome than the other, IPF-LASSO might thus be considered for prediction model building. This is a common scenario in clinical biomarker development: for example, we may have a small panel of protein markers identified based on strong prior biological knowledge and a profiling panel of whole-genome mRNA expression. Figure 1(d) for settings D, E, and F shows that, in IPF-LASSO, cross-validation is able to recognize which modality should be penalized more.

#### 3.2.2. Summary of All Simulation Results

**Figure 2:**
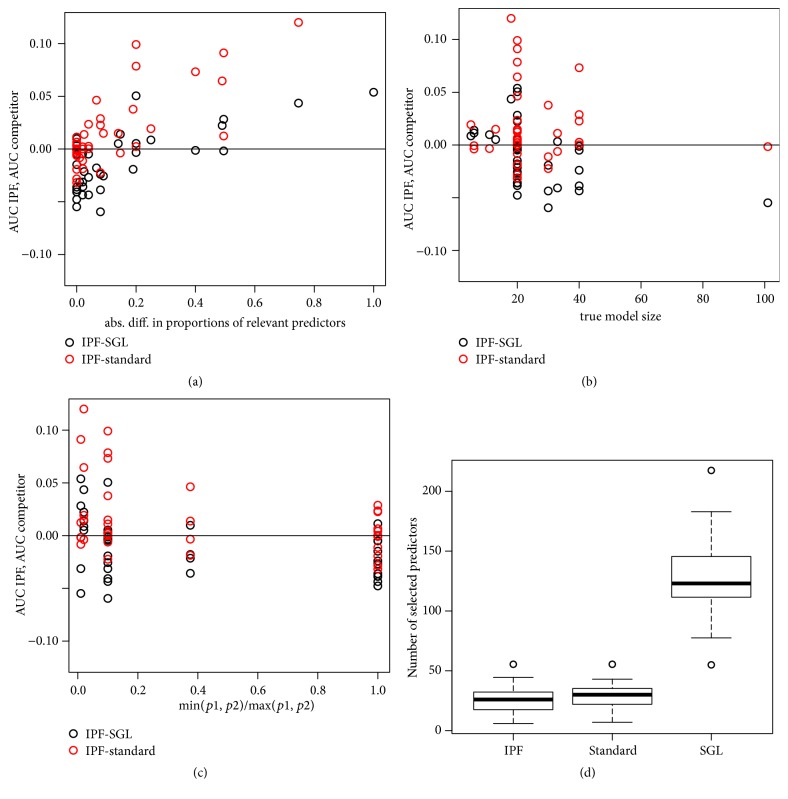
Panels (a), (b), and (c): difference Δ between the median AUC of IPF-LASSO and the median AUC of the standard LASSO (red points) and between the median AUC of IPF-LASSO and the median AUC of SGL (black points) against simulation parameters. A positive difference indicates better performance of IPF-LASSO. Each point on the scatterplots represents one of the 6 + 33 = 39 simulation settings. Panel (a): Δ against the absolute difference |*p*_1_^*r*^/*p*_1_ − *p*_2_^*r*^/*p*_2_| between the proportions of relevant variables in the two modalities. Panel (b): Δ against the true model size *p*_1_^*r*^ + *p*_2_^*r*^. Panel (c): Δ against a measure of the relative size of the modalities: min⁡(*p*_1_, *p*_2_)/max⁡(*p*_1_, *p*_2_). Panel (d): Median number of selected variables for IPF-LASSO, standard LASSO, and SGL. Each boxplot represents the values obtained for the 33 + 6 = 39 settings.

To further understand the method performance with respect to the two modalities in the simulations, we perform a large number of simulations using further parameter settings as summarized in Figure 2 (see Supplementary Table  1 for the corresponding parameter settings). We compile all results from the 6 main simulations and 33 additional settings, with one dot in each of panels (a), (b), and (c) of Figure 2 representing one simulation setting. Panel (a) shows the difference in median AUC over *B* = 100 simulation runs between IPF-LASSO and the standard LASSO (red dots), or SGL (black dots). Panel (b) shows the difference in median AUC against the true model size (true number of predictors). Panel (c) contains the difference in median AUC against a measure of the relative size of the modalities: min⁡(*p*_1_, *p*_2_)/max⁡(*p*_1_, *p*_2_). Panel (d) displays the distribution of the numbers of variables selected by the three methods.

Panel (a) in Figure 2 suggests that the larger the difference in proportions of truly relevant predictors between the two modalities (|*p*_1_^*r*^/*p*_1_ − *p*_2_^*r*^/*p*_2_|) is, the better IPF-LASSO performs compared to the standard LASSO and SGL. The simulation results in the 33 additional settings strengthen the conclusion of the main simulation. Panel (b) suggests that SGL works better than IPF-LASSO when there are large numbers of true variables, probably because it tends to select a lot more variables on average, as shown in panel (d). More precisely, SGL selects more than 100 variables most of the time, whereas IPF-LASSO selects only about 30 variables on average. When the true model size is small, IPF-LASSO is slightly better. The ratio min⁡(*p*_1_, *p*_2_)/max⁡(*p*_1_, *p*_2_) between smaller modality size and larger modality size displayed in panel (c) seems to impact the relative performance of IPF-LASSO and the standard LASSO: the smaller the ratio, the better the IPF-LASSO compared to the standard LASSO. We note, however, that this apparent association may be confounded by the proportion of relevant variables depicted in panel (a).

**Figure 3:**
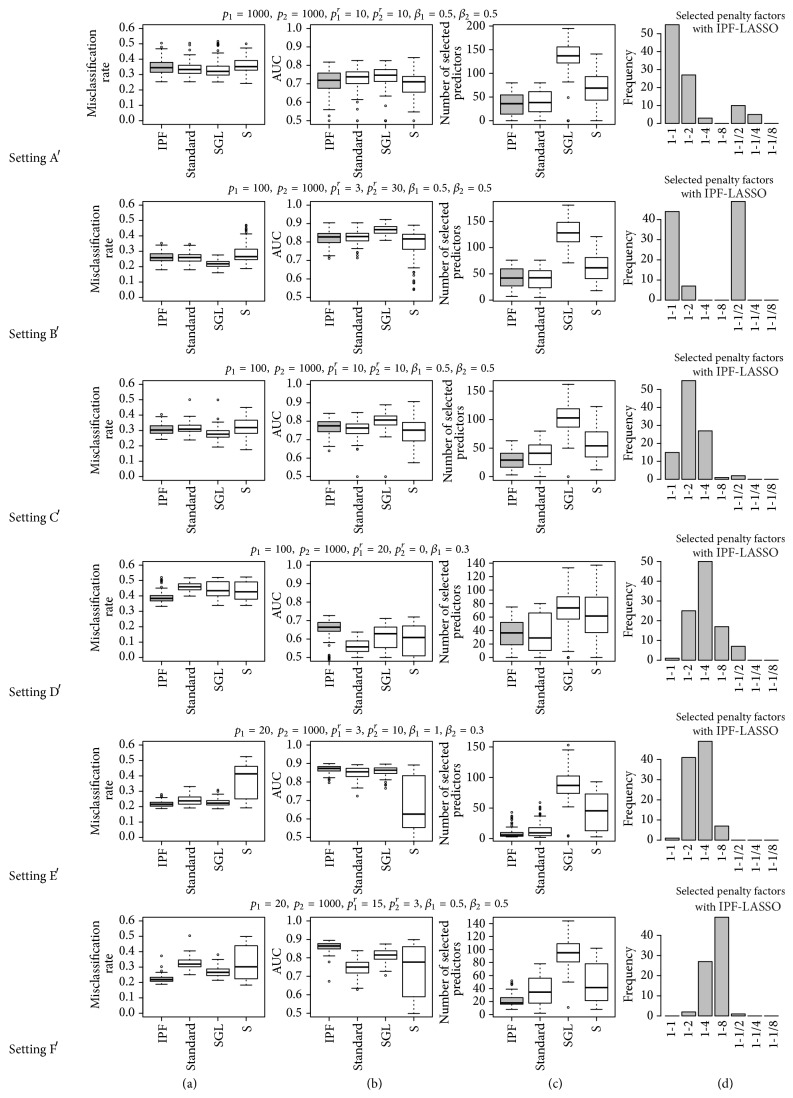
Results for settings A′ to F′ (with correlation): misclassification rate on test set (a), AUC on test set (b), number of selected variables (c), and penalty factors selected by IPF (d).

The results of settings A′ to F′ (with correlation) are very similar to the results of settings A to F, as can be seen from Figure 3. Correlation—at least the form of correlation considered here—does not seem to substantially affect our conclusions drawn with uncorrelated data.

## 4. Real Data Examples

### 4.1. TCGA Data

#### 4.1.1. Data

We use publicly available data on acute myeloid leukemia (AML) from The Cancer Genome Atlas [3]. Among those collected in this study, we consider three modalities, one low-dimensional (clinical data) and two high-dimensional, namely, microarray gene expressions and somatic copy number alterations. The outcome is the overall survival time (in month) of the patients, a possibly censored time-to-event response. The data are available from The Cancer Genome Atlas repository, with reference name LAML.

Clinical variables are the age, the percentage of blast cells in bone marrow, the white blood cell count per mm^3^ (continuous variables), and the sex. Preliminary analyses (not shown) show that, for these variables, the proportional hazards assumption is acceptable. One of the two molecular modalities consists of 19,798 microarray gene expression measurements from Affymetrix-U133 Plus 2. In the TCGA repository, they are available at different processing stages. Here we use the preprocessed data (level 3). As a second modality, we consider the copy number alterations obtained using Affymetrix SNP array 6.0. We download the data from the repository following the procedure of Zhao et al. [6]. We obtain 21,952 features, with values in {−2, −1,0, 1,2}. Each variable is coded as two dummy variables, one for negative alterations (values −2 and −1) and one for positive alterations (values 1 and 2). The absence of alteration (value 0) is used as the reference level. This modality includes 43,904 variables.

The clinical, gene expression, and copy number modalities have 200, 173, and 191 patients, respectively, which results in a total of 163 subjects with data for all three modalities. Since in the original study the data are not separated into training and validation sets, we generate this split randomly. More precisely, we use around 2/3 of the observations (109) for training our models (training set) and the rest (64) to compute their prediction ability (validation set). In our analysis, we consider 100 such random splits and present the average results.

#### 4.1.2. Results

**Figure 4:**
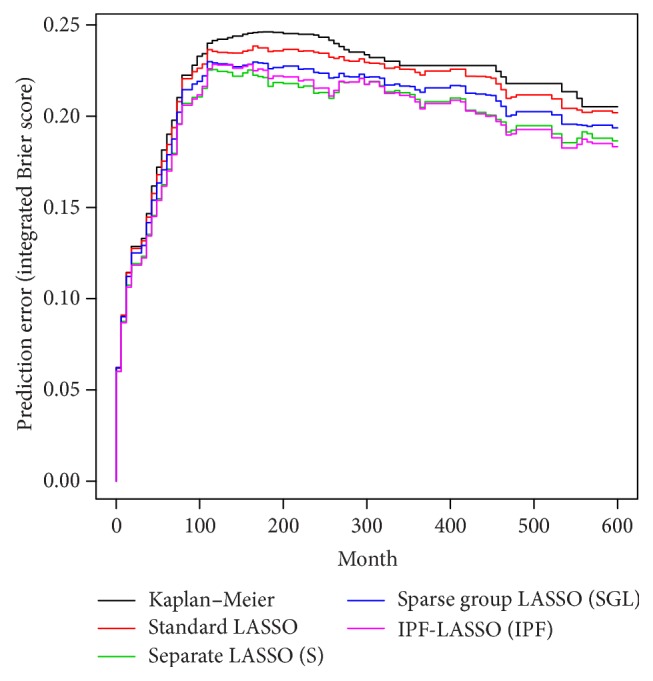
AML data. Prediction error curves computed up to 5 years for the models obtained by standard LASSO (red line), S (green line), SGL (blue line), and IPF-LASSO (purple line). The black line represents the prediction error obtained with the null model (no variables).

We compare the prediction abilities of Cox proportional hazards models obtained with the four different approaches (IPF, standard, SGL, and S) for the AML data. We also include the results from the nonparametric Kaplan-Meier method (the null model).  Figure 4 shows the prediction error curves based on the time-dependent Brier score [24] for the obtained models. A lower Brier score indicates a better prediction. More precisely, the prediction curves in Figure 4 report for each time point the mean of the 100 Brier scores computed at that time in all the training/validation sets splits considered in our analysis.

In this example, we note that IPF-LASSO (purple line) performs better than the standard LASSO and SGL (red and blue lines, resp.). Interestingly, if we apply LASSO separately to the different modalities (green line), the results are comparable to IPF-LASSO. The comparison in terms of prediction ability can be also performed numerically by evaluating the integrated Brier score (IBS), which summarizes the aforementioned curves into a single index. In this example, the standard LASSO has the worst performance (average IBS = 0.211), not much better than that of the null model (average IBS = 0.217). SGL performs a bit better (average IBS = 0.203) but worse than IPF-LASSO and S, which have both an average IBS equal to 0.196. In terms of sparsity, although IPF-LASSO and S have similar performance in terms of Brier score, IPF-LASSO produces much sparser models than S. On average, the numbers of variables in IPF-LASSO models and in S models are 7.3 and 13.7, respectively, with the standard LASSO between these two values (10.2). Not surprisingly, SGL (using the default value for the tuning parameter, *α* = 0.95) leads to substantially larger models, with an average of 53.64 variables.

### 4.2. Breast Cancer Data

#### 4.2.1. Data

Hatzis et al. [2] study the performance of a genomic signature for response and survival following taxane-anthracycline chemotherapy in patients with ERBB2-negative breast cancer. The outcome of interest is the (censored) distant relapse free survival time, that is, the time interval between the initial diagnosis biopsy and either the diagnosis of distant metastasis or death. The data are publicly available from the Gene Expression Omnibus repository with reference number GSE25066. This dataset contains two modalities, one low-dimensional (clinical data) and one high-dimensional (microarray gene expression data) modality.

Among the available clinical variables, we select age (continuous), nodal status (4 categories), tumor size (4 categories), grade (3 categories), estrogen receptor (binary), and progesterone receptor (binary) as described in De Bin et al. [8]. The second (high-dimensional) modality consists of 22,283 microarray gene expression measurements measured with Affymetrix-U133A GeneChip. We use the data preprocessed and normalized in the original paper [2] but without applying their first preselection step; that is, we consider the information of all the available probe sets.

The dataset consists of a training set used for training the genomic signature with 310 patients and a validation set with 198 patients. They include 66 and 45 patients who died (events), respectively. After removing subjects with missing data, there are 283 (58 events) and 182 (41 events) subjects in the training and validation datasets, respectively.

#### 4.2.2. Main Results

**Figure 5:**
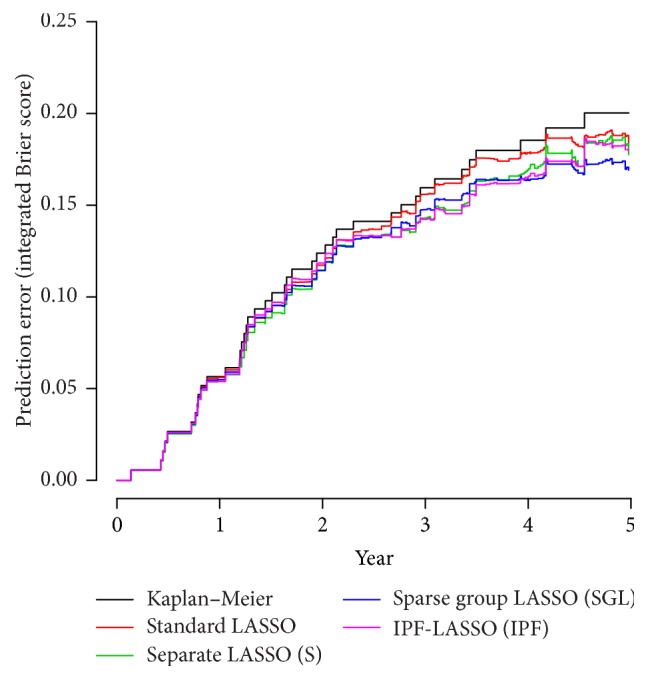
Breast cancer data. Prediction error curves computed up to 6 years for the models obtained by LASSO (red line), LASSO applied separately to the three modalities (green line), sparse group LASSO (blue line), and IPF-LASSO (purple line). The black line represents the results obtained with the null model (no variables).

Similar to the previous real dataset analysis, here we compare the Brier scores generated from the Cox proportional hazards models obtained with the four methods, that is, IPF-LASSO, SGL, S, and the standard LASSO, together with the null model from the nonparametric Kaplan-Meier method.  Figure 5 reports the Brier score computed on the validation set using the model trained on the training set. As shown in Figure 5, IPF-LASSO, SGL, and S perform very similarly overall. They are almost identical in predicting events happening in less than 3 years. They are better than the standard LASSO and the null model with Kaplan-Meier. For events between 3 and 4 years, IPF-LASSO and S seem slightly better than SGL; for events beyond 4 years, especially after 4.5 years, SGL appears better. However, these differences are minimal. The Brier scores for methods IPF-LASSO, S, SGL, the standard LASSO, and the null model are 0.129, 0.127, 0.130, 0.134, and 0.136, respectively. In terms of sparsity, we note that IPF-LASSO produces the sparsest model with 10 variables, followed by the standard LASSO with 20 variables and S with 27 variables. SGL generates a huge model containing 1084 variables.

#### 4.2.3. Flexible Choice of Penalty Factors

One advantage of IPF-LASSO is the possibility of flexibly choosing different weights for the different modalities. In this example, we observe that the cross-validation procedure selects the penalty factors (1,32), which penalize the molecular modality much more than the clinical modality. This is not a surprise, as several papers have shown the absence of a large added predictive value of microarray gene expression data in the case of breast cancer [8].

**Figure 6:**
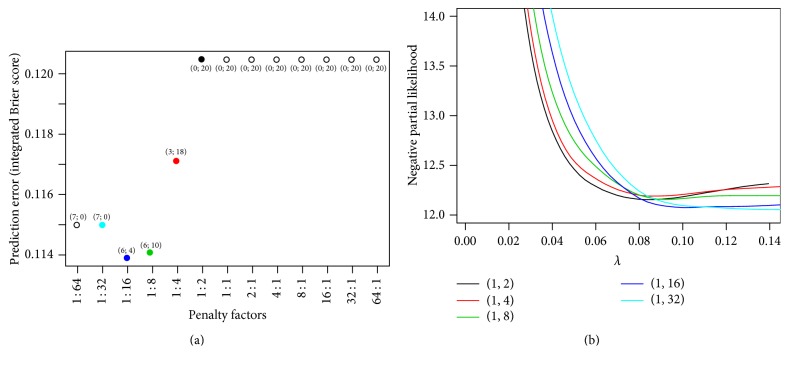
Breast cancer data. (a) Integrated Brier score obtained with IPF-LASSO for different choices of penalty factors. The numbers associated with the points are the numbers of selected clinical and molecular variables, respectively. For example, “(3-18)” indicates that for the penalty factors (1,4) the selected model includes 3 clinical variables and 18 molecular variables. (b) The negative partial likelihood against the parameter *λ* for different penalty factors. The colors of the curves are the colors of the corresponding points in (a).

The best model from IPF-LASSO (with penalty factor (1,32)) selects only clinical variables: age, estrogen receptor, tumor size, number of nodes, and tumor grade, yielding a total of 7 coefficients (since the number of nodes is represented by 3 coefficients). If we reduce the penalty factor of the molecular data, some gene expression variables get included into the model. For example, when decreasing the penalty factor of the molecular modality from 32 to 16 (i.e., the molecular modality is penalized 16 times more than the clinical modality), the gene expression probe sets 203153_at, 203860_at, 217769_s_at, and 219097_x_at enter the model and the clinical variable tumor grade is excluded. At this time, we obtain a small improvement in the prediction ability of the model on validation data (see Figure 6(a)). Decreasing the penalty factor of the molecular modality to 8 leads to more gene expression variables entering in the model, whereas the prediction ability on validation data is similar to that with penalty factor 16. A further decrease to 4 results in the exclusion of one more clinical variable (the number of lymph nodes) and the inclusion of more molecular variables. The prediction ability of the model, however, decreases, supporting the idea of the strong relevance of clinical variables. Note that the size of the model increases from 7 clinical variables only (the best model with penalty factor of 32 for the clinical modality) to 21 with penalty factor 4. If we further decrease the relative penalty for the molecular modality, IPF-LASSO does not select any clinical variable. If not adequately favored, the clinical variables “get lost” among the molecular ones due to the vastly different sizes of the two modalities. As a consequence, the prediction ability of the model worsens. For example, with the penalty factors (1,2), the integrated Brier score increases to 0.120, a value close to that obtained for the standard LASSO.

Figure 6(b) displays the cross-validated negative partial likelihood (based on training data) against the parameter *λ* for the penalty factors (1,2), (1,4), (1,8), (1,16), and (1,32). Note that the colors of the curves are the colors of the corresponding points in the plot of the left panel. These curves confirm that, according to cross-validation, the best model is obtained for (1,32) (the curves for more extreme penalty factors, which we omit for visibility purposes, have a higher minimum than those displayed in Figure 6(b)). The curves also allow for visualizing the two-dimensional optimization process performed by cross-validation: IPF-LASSO selects the penalty factors and the value of *λ* optimizing the criterion, that is, the point with the smallest *y*-value across all curves.

#### 4.2.4. Results with Binary Outcome

In addition to modelling the distant relapse-free survival time, a secondary goal of this study is to distinguish the patients with a pathological complete response (RCB-I) from those with a significant residual disease (RCB-II/RCB-III). Here, the pathological response is a binary outcome. We now apply the four approaches considered previously with logistic regression and use the area under the ROC curve (AUC) as a performance metric for the methods. In contrast to the Brier score, a larger value of AUC corresponds to better prediction performance. The AUC values for IPF-LASSO, S, SGL, and the standard LASSO are 0.663, 0.712, 0.722, and 0.653, respectively. Regarding the model sparsity, IPF-LASSO and S select a comparable number of variables (50 and 46, resp.), while the standard LASSO leads to the sparsest model (38 variables). Again SGL provides a much larger model with 1128 variables. Please note that this unfavorable result of our method is not contradictory per se with the simulation results, since a real dataset is but a point in the space of all possible datasets, and the performance of methods is highly variable across datasets [25]. In this paper, we make the choice to honestly report this unfavorable result and not to report only the results that make our method look better, following Rule  4 (“do not fish for datasets”) of the good practice recommendations by Boulesteix [26].

## 5. Discussion

In this paper, we addressed an important question in biomedical research, namely, how to integrate multiple (possibly correlated) data modalities with different sizes and different relevancies to the outcome, with the aim of generating a sparse prediction model. We proposed an *L*_1_-penalized regression method, IPF-LASSO, that penalizes the data modalities differently. IPF-LASSO is flexible in determining the penalty factors—they can be chosen in a completely data-driven manner by cross-validation or specified by user. IPF-LASSO works with continuous, binary, or survival dependent variables; and predictor variables can be continuous, categorical, and a mixture of both. IPF-LASSO is implemented in our R package ipflasso but could in principle be integrated within any package implementing *L*_1_-penalized regression, such as glmnet. Most importantly, being directly based on LASSO, our approach has two major advantages: its conceptual simplicity within a well-established framework and its computational* transportability* allowing easy application of the resulting prediction rules by other researchers.

Simulation studies have demonstrated that IPF-LASSO has better prediction performance compared to competitors (standard LASSO, separate LASSO models, and sparse group LASSO), when the two data modalities are different in terms of relevance for prediction, and performs slightly worse if the modalities are similar. More importantly, in both simulations and real case studies, IPF-LASSO is shown to generate much more parsimonious models than sparse group LASSO, which is a much desired property from a practical perspective.

In principle, IPF-LASSO is designed for any number *M* of modalities. It assigns one penalty factor to each modality, with its value controlling how much a modality is penalized when fitting the model. In practice, however, the choice of the penalty factors is a computational bottle-neck, since the computation time required by full cross-validation grows exponentially with *M*. With today's computational capacities, full cross-validation is manageable only for up to, say, *M* = 4 modalities. In contrast, sparse group LASSO has one unique parameter for all modalities; hence, it is not able to distinguish differences in modalities. This makes it less flexible but more appropriate to handle large numbers of modalities. Note that the good performance of sparse group LASSO observed in simulations comes at a price of generating substantially larger models, which may not be practical in real-life applications. In addition, having different penalty factors in IPF-LASSO allows for the incorporation of prior biological knowledge or practical concerns. To address the computational cost induced by the choice of the penalty factors for large *M*, alternatives to our grid search cross-validation approach may be considered in the future, for example, based on empirical Bayes procedures [20], on model selection criteria such as the Akaike information criterion (AIC) or Bayesian information criterion (BIC), or using the approach inspired from adaptive LASSO [14] adopted by Ternès et al. [27] in the specific case of treatment-biomarker interactions.

One common issue for all variations of LASSO, including IPF-LASSO, is instability. Small changes of the dataset may lead to big changes of the selected model. Stability can be investigated using resampling methods, as suggested under the name “stability selection” [28]. Such methods, which are increasingly gaining attention [29], can be directly applied to IPF-LASSO as well. Going beyond the scope of this work, further improvement from IPF-LASSO may be considered. For example, one may consider introducing additional *L*_2_ penalty term(s), yielding “elastic net-” like methods [30].

## Supplementary Material

The table displays the parameters of the additional simulation settings. See the results in Subsection 3.2.2.

## Figures and Tables

**Table 1:** Combinations of *p*_1_, *p*_2_, *p*_1_^*r*^, *p*_2_^*r*^, *β*_1_, and *β*_2_ used for the *main design*. All other parameters are fixed (*n* = 100, *τ* = 0.5, Σ = **I**_*p*_1_+*p*_2__). For each setting, *B* = 100 datasets are successively generated.

	*p*_1_	*p*_2_	*p*_1_^*r*^	*p*_2_^*r*^	*β*_1_	*β*_2_
Setting A	1000	1000	10	10	0.5	0.5
Setting B	100	1000	3	30	0.5	0.5
Setting C	100	1000	10	10	0.5	0.5
Setting D	100	1000	20	0	0.3	
Setting E	20	1000	3	10	1	0.3
Setting F	20	1000	15	3	0.5	0.5
