# Supplementary material for: IPF-LASSO: Integrative L 1-Penalized Regression with Penalty Factors for Prediction Based on Multi-Omics Data
Source: Comput Math Methods Med. 2017 May 4;2017:7691937. doi: 10.1155/2017/7691937 (PMC5435977; doi:10.1155/2017/7691937)
Supplement: Supplementary file 1 — The table displays the parameters of the additional simulation settings. See the results in Subsection 3.2.2. [file 7691937.f1.pdf]

	p_1	p_2	p_1^r	p_2^r	β_1	β_2
setting A	1000	1000	10	10	0.5	0.5
	100	1000	0	20		0.5
	100	1000	0	20		1
	100	1000	0	40		0.5
setting C	100	1000	10	10	0.5	0.5
	100	1000	10	20	0.5	0.5
setting D	100	1000	20	0	0.3	
	100	1000	20	0	0.5	
	100	1000	20	0	1	
	100	1000	20	10	0.5	0.5
setting B	100	1000	3	30	0.5	0.5
	100	1000	3	30	1	0.3
	100	1000	40	0	0.5	
	100	100	10	10	0.5	0.5
	20	1000	10	10	0.5	0.5
setting F	20	1000	15	3	0.5	0.5
setting E	20	1000	3	10	1	0.3
	20	1000	3	3	1	1
	20	1000	5	0	1	
	20	2000	0	20		0.5
	20	2000	10	10	0.5	0.5
	20	2000	10	10	1	0.3
	20	2000	1	100	0.5	0.5
	20	2000	20	0	0.5	
	300	800	0	20		0.5
	300	800	10	10	0.5	0.5
	300	800	20	0	0.5	
	300	800	3	8	0.8	0.8
	500	500	10	10	0.3	0.8
	500	500	10	10	0.3	1.5
	500	500	10	10	0.4	0.7
	500	500	10	10	0.5	0.5
	500	500	20	0	0.5	
	500	500	20	0	1	
	500	500	20	10	0.5	0.5
	500	500	20	20	1	0.3
	500	500	3	3	1	1.5
	500	500	40	0	0.3	
	500	500	40	0	0.5	

Supplementary Table 1: Parameter settings for the main design (A to F) and the additional simulations.
